# Supplementary material for: Multi-tissue transcriptome-wide association study identifies eight candidate genes and tissue-specific gene expression underlying endometrial cancer susceptibility
Source: Commun Biol. 2021 Oct 21;4:1211. doi: 10.1038/s42003-021-02745-3 (PMC8531339; doi:10.1038/s42003-021-02745-3)
Supplement: Supplementary file 2 — Supplementary Information [file 42003_2021_2745_MOESM2_ESM.pdf]

Multi-tissue transcriptome-wide association study identifies eight candidate genes and tissue-specific gene expression underlying endometrial cancer susceptibility

Pik Fang Kho, Xuemin Wang, Gabriel Cuellar Partida, Thilo Dörk, Ellen L. Goode[4], Diether Lambrechts, Rodney J. Scott, Endometrial Cancer Association Consortium, Amanda B. Spurdle, Tracy A. O'Mara, Dylan M. Glubb.

Supplementary Figure

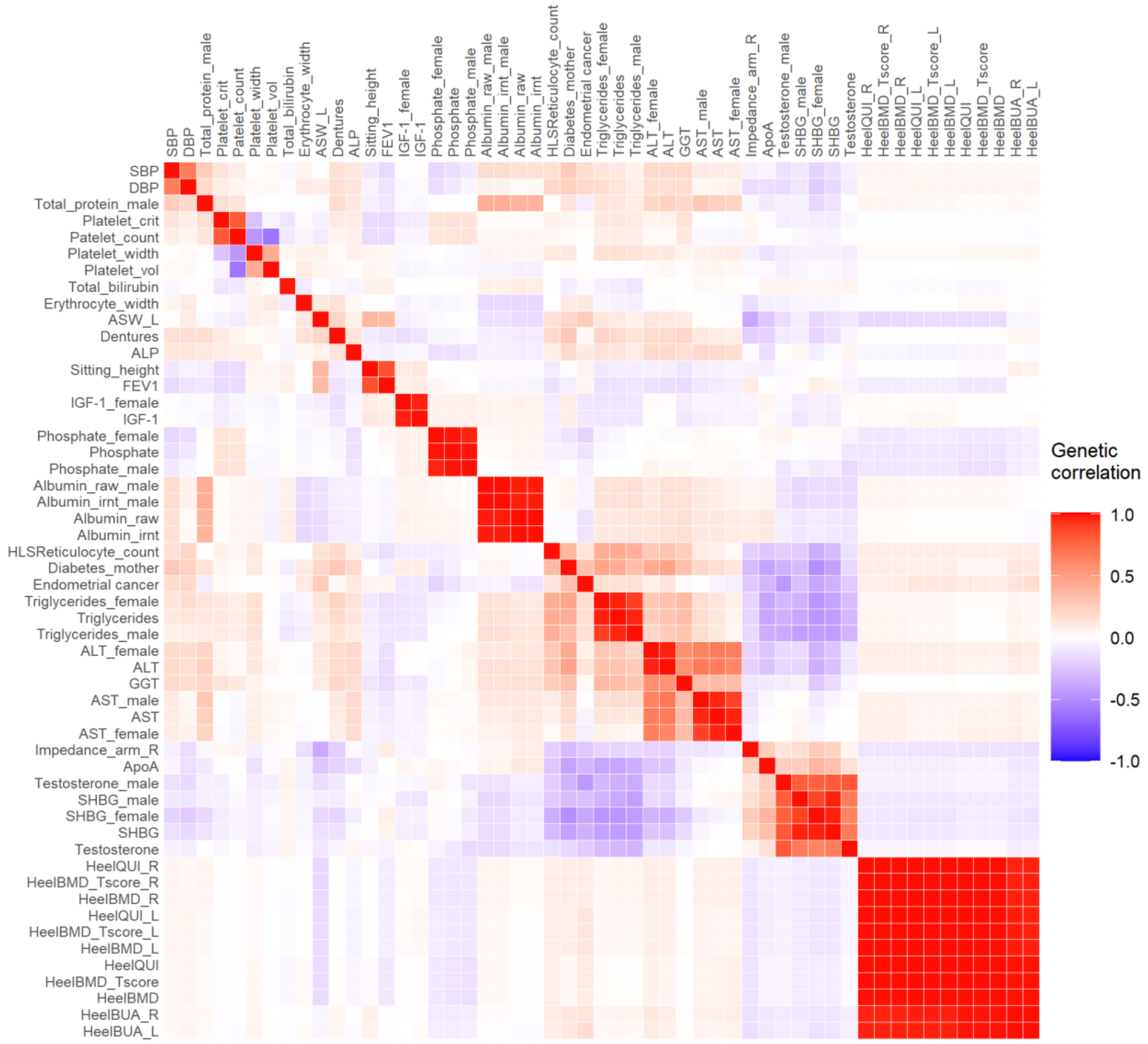

Supplementary Figure 1. Pair-wise genetic correlation of endometrial cancer and PheWAS traits. The intensity of the color shows the magnitude of the genetic correlation between traits, where intense red color indicates a strong positive correlation and intense blue color indicates a strong negative correlation.

Supplementary Tables

Supplementary Table 1. Genes associated with endometrioid endometrial cancer risk as identified by S-MultiXcan and colocalization

Locus	Gene	N_{tissue}	P	Z_{min}	Z_{max}	Z_{mean}	Z_{SD}	$PP_{Adip. Sub.}$	$PP_{Adip. Vis.}$	PP_{Ovary}	PP_{Vagina}	PP_{Uterus}	PP_{WB}
**6q22.31**	***HEY2***	**1**	**2.46E-09**	**-5.96**	**-5.96**	**-5.96**	**NA**	**0.00**	**0.00**	**0.91**	**0.01**	**0.02**	**0.00**
15q21.2	*GLDN*	4	1.27E-09	-1.80	3.98	0.11	2.65	0.00	0.00	0.01	0.02	0.00	0.00
**15q21.2**	***CYP19A1***	**2**	**1.82E-09**	**4.93**	**5.64**	**5.29**	**0.50**	**0.35**	**0.02**	**0.00**	**0.01**	**NA**	**0.85**
**17q21.32**	***SKAP1***	**2**	**5.95E-09**	**-6.11**	**0.19**	**-2.96**	**4.46**	**0.01**	**0.01**	**0.01**	**0.01**	**0.06**	**0.94**
17q21.32	*SNX11*	4	3.81E-07	3.09	5.20	4.45	0.93	0.03	0.03	0.04	0.12	0.04	0.03

N_{tissue}: number of tissues available for this gene; Z_{min}: minimum Z score from single tissue S-PrediXcan result; Z_{max}: maximum Z score from single tissue S-PrediXcan result; Z_{mean}: mean Z score from single tissue S-PrediXcan result; Z_{SD}: standard deviation of Z score from single tissue S-PrediXcan result; PP: posterior probability that endometrial cancer risk and eQTL variants from respective tissue colocalize; Adip. Sub.: Subcutaneous adipose; Adip. Vis.: Visceral omentum adipose; WB: Whole blood. Bonferroni corrected P value is 3.8×10^{-6} accounting for 13,182 genes tested. Bolded findings have evidence of colocalization (PP>0.8) in at least relevant tissue.

Supplementary Table 2. Genes associated with endometrioid endometrial cancer risk as identified by MR-JTI

Tissue	Locus	Gene	JTI		MR-JTI	
			Z-score	P-value	Beta	95% CI
Subcutaneous Adipose	15q21.2	*CYP19A1*	7.21	5.75E-13	-0.01	(-0.20,0.22)
	17q21.32	*AC004477.1*	4.89	1.03E-06	0.01	(-0.31,0.25)
	**17q21.32**	***SNX11***	**4.72**	**2.33E-06**	**0.28**	**(0.01,0.58)**
	17q21.32	*AC004477.3*	-4.67	3.07E-06	-0.26	(-0.50,0.07)
Visceral Omentum Adipose	15q21.2	*CYP19A1*	6.79	1.15E-11	0.10	(-0.08,0.29)
	17q21.32	*AC004477.1*	4.97	6.84E-07	-0.01	(-0.32,0.34)
	**17q21.32**	***SNX11***	**4.73**	**2.28E-06**	**0.61**	**(0.39,0.86)**
Ovary	6q22.31	*HEY2*	-5.96	2.47E-09	0.05	(-0.29,0.20)
	17q21.32	*AC004477.1*	5.01	5.34E-07	0.04	(-0.30,0.26)
	15q15.1	*EIF2AK4*	4.63	3.72E-06	-0.09	(-0.17,0.41)
Vagina	**15q15.1**	***EIF2AK4***	**4.87**	**1.11E-06**	**0.18**	**(0.03,0.41)**
Whole Blood	15q21.2	*CYP19A1*	5.25	1.50E-07	-0.12	(-0.34,0.10)
	17q21.32	*SKAP1*	-4.61	4.09E-06	0.07	(-0.04,0.45)
	**17q21.32**	***SNX11***	**4.57**	**4.79E-06**	**0.42**	**(0.15,0.70)**

Bonferroni corrected P values were: 3.7×10^{-6} (13,526 genes tested) in $JTI_{subcutaneous\ adipose}$; 3.9×10^{-6} (12,949 genes tested) in $JTI_{visceral\ omentum\ adipose}$, 5.8×10^{-6} (8,615 genes tested) in JTI_{Ovary}, 7.0×10^{-6} (7,191 genes tested) in JTI_{vagina} and 4.9×10^{-6} (10,140 genes tested) in $JTI_{whole\ blood}$. Bolded findings have evidence of causal support in MR-JTI analyses.

Supplementary Table 3. Phenotypes associated with endometrial cancer susceptibility genes

Phenotypic category	Trait	Gene	Subcutaneous Adipose			Visceral Omentum Adipose			Whole Blood		
			B_{SMR} (se)	P_{SMR}	P_{HEIDI}	B_{SMR} (se)	P_{SMR}	P_{HEIDI}	B_{SMR} (se)	P_{SMR}	P_{HEIDI}
**Anthropometric**	Impedance of arm (right)	*EVI2A*	-	-	-	-	-	-	3.16<br>-0.76	2.93E-05	0.05
	Sitting height	*EIF2AK4*	-	-	-	0.17<br>-0.04	1.16E-05	0.18	-	-	-
**Bone health**	Ankle spacing width (right)	*SNX11*	-	-	-	-0.21<br>-0.05	2.60E-05	0.08	-0.32<br>-0.07	1.38E-05	0.06
	Heel bone mineral density (BMD)	*CYP19A1*	-	-	-	-	-	-	0.02<br>0.00	2.73E-07	0.07
	Heel bone mineral density (BMD) (left)	*CYP19A1*	-	-	-	-	-	-	0.02<br>0.00	4.74E-07	0.12
	Heel bone mineral density (BMD) (right)	*CYP19A1*	-	-	-	-	-	-	0.02<br>0.00	9.76E-07	0.18
	Heel bone mineral density (BMD) T-score, automated	*CYP19A1*	-	-	-	-	-	-	0.14<br>-0.03	3.51E-07	0.07
	Heel bone mineral density (BMD) T-score, automated (left)	*CYP19A1*	-	-	-	-	-	-	0.19<br>-0.04	4.55E-07	0.13
	Heel bone mineral density (BMD) T-score, automated (right)	*CYP19A1*	-	-	-	-	-	-	0.17<br>-0.03	9.83E-07	0.18
	Heel broadband ultrasound attenuation (left)	*CYP19A1*	-	-	-	-	-	-	2.85<br>-0.57	5.52E-07	0.09
	Heel broadband ultrasound attenuation (right)	*CYP19A1*	-	-	-	-	-	-	2.54<br>-0.53	1.56E-06	0.11
		CYP19A1	-	-	-	-	-	-	2.46	3.51E-07	0.07

Phenotypic category	Trait	Gene	Subcutaneous Adipose			Visceral Omentum Adipose			Whole Blood		
			B_{SMR} (se)	P_{SMR}	P_{HEIDI}	B_{SMR} (se)	P_{SMR}	P_{HEIDI}	B_{SMR} (se)	P_{SMR}	P_{HEIDI}
	Heel quantitative ultrasound index (QUI), direct entry								-0.48		
	Heel quantitative ultrasound index (QUI), direct entry (left)	*CYP19A1*	-	-	-	-	-	-	3.28<br>-0.65	4.55E-07	0.13
	Heel quantitative ultrasound index (QUI), direct entry (right)	*CYP19A1*	-	-	-	-	-	-	3.01<br>-0.62	9.84E-07	0.18
	Mouth/teeth dental problems: Dentures	*SNX11*	-0.02<br>0.00	6.27E-06	0.14	-0.01<br>0.00	3.29E-06	0.30	-0.02<br>0.00	1.91E-06	0.35
	Phosphate (quantile) both_sexes	*CYP19A1*	-	-	-	-	-	-	-0.10<br>-0.02	1.32E-07	0.07
	Phosphate (quantile) female	*CYP19A1*	-	-	-	-	-	-	-0.09<br>-0.02	1.34E-05	0.23
	Phosphate (quantile) male	*CYP19A1*	-	-	-	-	-	-	-0.12<br>-0.02	2.68E-06	0.13
**Cardiovascular**	Apolipoprotein A (quantile) both_sexes	*EVI2A*	-	-	-	-	-	-	0.08<br>-0.02	2.80E-05	0.08
	Diastolic blood pressure, automated reading	*EIF2AK4*	-	-	-	-	-	-	-0.68<br>-0.14	1.36E-06	0.08
	Forced expiratory volume in 1-second (FEV1), predicted	*SNX11*	-	-	-	-	-	-	-0.02<br>0.00	1.77E-05	0.63
	Systolic blood pressure, automated reading	*EIF2AK4*	-	-	-	-	-	-	-1.27<br>-0.25	5.50E-07	0.06
	Triglycerides (quantile) both_sexes	*SNX11*	-	-	-	-	-	-	0.07<br>-0.01	3.83E-09	0.19
	Triglycerides (quantile) female	*SNX11*	-	-	-	-	-	-	0.06	1.53E-05	0.36

Phenotypic category	Trait	Gene	Subcutaneous Adipose			Visceral Omentum Adipose			Whole Blood		
			**B_{SMR} (se)**	**P_{SMR}**	**P_{HEIDI}**	**B_{SMR} (se)**	**P_{SMR}**	**P_{HEIDI}**	**B_{SMR} (se)**	**P_{SMR}**	**P_{HEIDI}**
									-0.01		
	Triglycerides (quantile) male	*SNX11*	0.06<br>-0.01	1.54E-05	0.20	0.05<br>-0.01	8.94E-06	0.28	0.07<br>-0.02	4.97E-06	0.31
**Diabetes**	IGF-1 (quantile) both_sexes	*CYP19A1*	-	-	-	-	-	-	-0.06<br>-0.01	1.81E-05	0.06
	IGF-1 (quantile) female	*CYP19A1*	-	-	-	-	-	-	-0.09<br>-0.02	1.50E-05	0.15
	Illnesses of mother: Diabetes	*SNX11*	-	-	-	0.01<br>0.00	2.28E-05	0.36	0.01<br>0.00	1.97E-05	0.29
**Hematopoiesis**	High light scatter reticulocyte count	*EIF2AK4*	0.00<br>0.00	3.11E-05	1.00	-	-	-	-	-	-
	Mean platelet (thrombocyte) volume	*EVI2A*	-	-	-	-	-	-	-0.10<br>-0.02	4.49E-06	0.25
	Platelet count	*EVI2A*	-	-	-	-	-	-	7.21<br>-1.38	1.92E-07	0.43
	Platelet crit	*EVI2A*	-	-	-	-	-	-	0.00<br>0.00	1.72E-05	0.19
	Platelet distribution width	*EIF2AK4*	-	-	-	-	-	-	0.04<br>-0.01	6.75E-07	0.36
	Red blood cell (erythrocyte) distribution width	*EIF2AK4*	-	-	-	0.05<br>-0.01	2.03E-06	0.09	-	-	-
**Liver function**	Alanine aminotransferase (quantile) both_sexes	*EIF2AK4*	-	-	-	-0.06<br>-0.01	1.69E-07	0.08	-	-	-
	Alanine aminotransferase (quantile) female	*EIF2AK4*	-	-	-	-0.07<br>-0.02	1.74E-06	0.20	-	-	-

Phenotypic category	Trait	Gene	Subcutaneous Adipose			Visceral Omentum Adipose			Whole Blood		
			B_{SMR} (se)	P_{SMR}	P_{HEIDI}	B_{SMR} (se)	P_{SMR}	P_{HEIDI}	B_{SMR} (se)	P_{SMR}	P_{HEIDI}
	Albumin (g/L) both_sexes	*EIF2AK4*	-0.14<br>-0.02	2.84E-09	0.49	-0.16<br>-0.03	3.78E-07	0.47	-	-	-
	Albumin (g/L) male	*EIF2AK4*	-0.17<br>-0.03	1.25E-07	0.17	-0.20<br>-0.04	3.29E-06	0.31	-	-	-
	Albumin (quantile) both_sexes	*EIF2AK4*	-0.05<br>-0.01	2.11E-09	0.48	-0.06<br>-0.01	3.23E-07	0.45	-	-	-
	Albumin (quantile) male	*EIF2AK4*	-0.06<br>-0.01	8.33E-08	0.16	-0.08<br>-0.02	2.58E-06	0.30	-	-	-
	Alkaline phosphatase (quantile) both_sexes	*EVI2A*	-	-	-	-	-	-	-0.09<br>-0.02	1.12E-05	0.68
	Aspartate aminotransferase (quantile) both_sexes	*EIF2AK4*	-0.05<br>-0.01	1.91E-10	0.06	-0.07<br>-0.01	9.33E-08	0.31	-	-	-
	Aspartate aminotransferase (quantile) both_sexes	*EVI2A*	-	-	-	-	-	-	-0.10<br>-0.02	1.11E-06	0.06
	Aspartate aminotransferase (quantile) female	*EIF2AK4*	-	-	-	-0.07<br>-0.01	4.53E-06	0.19	-	-	-
	Aspartate aminotransferase (quantile) male	*EIF2AK4*	-0.06<br>-0.01	1.05E-06	0.11	-0.07<br>-0.02	1.25E-05	0.17	-	-	-
	Gamma glutamyltransferase (quantile) both_sexes	*EIF2AK4*	-0.04<br>-0.01	6.26E-07	0.09	-0.04<br>-0.01	8.94E-06	0.24	-	-	-
	Total bilirubin (quantile) both_sexes	*SNX11*	-	-	-	-	-	-	-0.04<br>-0.01	3.04E-05	0.31
	Total protein (quantile) male	*EIF2AK4*	-0.05<br>-0.01	9.04E-06	0.31	-	-	-	-	-	-
**Sex hormones**	SHBG (quantile) both_sexes	*EVI2A*	-	-	-	-	-	-	0.17	2.66E-09	0.31

Phenotypic category	Trait	Gene	Subcutaneous Adipose			Visceral Omentum Adipose			Whole Blood		
			**B_{SMR} (se)**	**P_{SMR}**	**P_{HEIDI}**	**B_{SMR} (se)**	**P_{SMR}**	**P_{HEIDI}**	**B_{SMR} (se)**	**P_{SMR}**	**P_{HEIDI}**
									-0.03		
	SHBG (quantile) both_sexes	*SNX11*	-	-	-	-	-	-	-0.05<br>-0.01	9.26E-06	0.14
	SHBG (quantile) female	*EVI2A*	-	-	-	-	-	-	0.16<br>-0.03	7.10E-07	0.2
	SHBG (quantile) male	*EVI2A*	-	-	-	-	-	-	0.22<br>-0.04	2.38E-08	0.1
	Testosterone (quantile) both_sexes	*CYP19A1*	-	-	-	-	-	-	-0.04<br>-0.01	1.92E-05	0.15
	Testosterone (quantile) both_sexes	*EVI2A*	-	-	-	-	-	-	0.06<br>-0.01	5.28E-06	0.47
	Testosterone (quantile) male	*CYP19A1*	-	-	-	-	-	-	-0.11<br>-0.02	1.95E-06	0.39
	Testosterone (quantile) male	*EVI2A*	-	-	-	-	-	-	0.16<br>-0.03	1.05E-06	0.13

Results presented are SMR associations that passed Bonferroni-correct P-value (P_{SMR}) and heterogeneity HEIDI P-value (P_{HEIDI}) threshold.

Supplementary Table 4. Genetic correlation between PheWAS traits and endometrial cancer

PheWAS traits	rg	SE	P value
**Testosterone (quantile) male**	-0.45	0.06	2.74E-14
**SHBG (quantile) female**	-0.38	0.06	1.45E-10
**SHBG (quantile) both_sexes**	-0.34	0.06	3.06E-09
**Apolipoprotein A (quantile) both_sexes**	-0.32	0.05	3.16E-09
**Impedance of arm (right)**	-0.24	0.04	9.71E-09
**Ankle spacing width (right)**	0.27	0.05	2.11E-07
**SHBG (quantile) male**	-0.28	0.05	2.90E-07
**Illnesses of mother: Diabetes**	0.33	0.09	1.00E-04
**Diastolic blood pressure, automated reading**	0.20	0.05	2.00E-04
**Systolic blood pressure, automated reading**	0.18	0.05	2.00E-04
**Testosterone (quantile) both_sexes**	-0.23	0.06	2.00E-04
**Heel broadband ultrasound attenuation (left)**	0.18	0.05	5.00E-04
**Triglycerides (quantile) both_sexes**	0.16	0.05	9.00E-04
Triglycerides (quantile) female	0.17	0.05	1.30E-03
Phosphate (quantile) female	-0.18	0.06	2.40E-03
Triglycerides (quantile) male	0.16	0.06	2.90E-03
Heel broadband ultrasound attenuation (right)	0.14	0.05	5.80E-03
Heel bone mineral density (BMD) (left)	0.13	0.05	7.50E-03
Heel bone mineral density (BMD) T-score, automated (left)	0.13	0.05	8.70E-03
Heel quantitative ultrasound index (QUI), direct entry (left)	0.13	0.05	8.70E-03
High light scatter reticulocyte count	0.14	0.06	0.01
Alanine aminotransferase (quantile) both_sexes	0.14	0.05	0.01
Phosphate (quantile) both_sexes	-0.12	0.05	0.01
Heel bone mineral density (BMD) T-score, automated	0.10	0.04	0.01
Heel quantitative ultrasound index (QUI), direct entry	0.10	0.04	0.01
Red blood cell (erythrocyte) distribution width	0.12	0.05	0.02

**PheWAS traits**	**rg**	**SE**	**P value**
Heel bone mineral density (BMD)	0.10	0.04	0.02
Alkaline phosphatase (quantile) both_sexes	0.11	0.05	0.02
Albumin (g/L) both_sexes	-0.12	0.05	0.02
Albumin (quantile) both_sexes	-0.12	0.05	0.02
IGF-1 (quantile) both_sexes	-0.09	0.04	0.02
IGF-1 (quantile) female	-0.11	0.05	0.03
Heel quantitative ultrasound index (QUI), direct entry (right)	0.09	0.05	0.06
Heel bone mineral density (BMD) T-score, automated (right)	0.09	0.05	0.06
Heel bone mineral density (BMD) (right)	0.09	0.05	0.07
Alanine aminotransferase (quantile) female	0.08	0.06	0.15
Sitting height	0.06	0.04	0.16
Total protein (quantile) male	-0.08	0.06	0.2
Aspartate aminotransferase (quantile) female	-0.07	0.06	0.21
Forced expiratory volume in 1-second (FEV1), predicted	0.05	0.05	0.29
Phosphate (quantile) male	-0.06	0.06	0.3
Aspartate aminotransferase (quantile) both_sexes	-0.05	0.05	0.33
Albumin (g/L) male	-0.05	0.06	0.41
Albumin (quantile) male	-0.05	0.06	0.45
Gamma glutamyltransferase (quantile) both_sexes	0.03	0.05	0.53
Mouth/teeth dental problems: Dentures	0.04	0.06	0.54
Platelet crit	0.03	0.05	0.57
Mean platelet (thrombocyte) volume	0.01	0.04	0.7
Platelet count	0.02	0.05	0.72
Total bilirubin (quantile) both_sexes	-0.02	0.1	0.82
Aspartate aminotransferase (quantile) male	-0.01	0.07	0.84
Platelet distribution width	0.00	0.05	0.93

PheWAS traits in bold passed Bonferroni correction (52 PheWAS traits tested, $P < 9.62\times10^{-4}$).

Supplementary Table 5. Drug targets of CYP19A1 as identified by Open Target Database

Drug	Indications	Status
Letrozole	Breast cancer	Approved
Anastrozole	Breast cancer	Approved
Exemestane	Breast cancer	Approved
Aminoglutethimide	Breast cancer, Cushing syndrome	Approved

Supplementary Table 6. Genes associated with endometrial cancer risk (FDR < 0.01), derived from JTI analyses

Tissue	Gene	Z-score	P value	FDR
Subcutaneous Adipose	*CYP19A1*	7.03	2.00E-12	2.70E-08
	SNX11	4.90	9.45E-07	4.63E-03
	AC004477.3	-4.89	1.03E-06	4.63E-03
	NPIPB6	4.78	1.74E-06	5.90E-03
	ATF7IP2	-4.63	3.70E-06	0.01
	AC004477.1	4.53	5.87E-06	0.01
	NF1	4.44	8.79E-06	0.02
	FPGS	-4.43	9.52E-06	0.02
	EIF3C	4.36	1.32E-05	0.02
	NFATC2IP	4.31	1.62E-05	0.02
	EVI2A	-4.20	2.65E-05	0.03
	HEY2	-4.12	3.84E-05	0.04
	AC109460.2	4.07	4.76E-05	0.05
	BRD3	-3.92	8.92E-05	0.09
	AC021755.2	3.89	9.83E-05	0.09
	UQCC1	-3.88	1.05E-04	0.09
	ATP2A1	3.86	1.15E-04	0.09
	AC064836.2	3.83	1.26E-04	0.09
	ABCC5	-3.83	1.29E-04	0.09
	ATXN2-AS	-3.82	1.36E-04	0.09
	AC005089.1	3.81	1.41E-04	0.09
	EEFSEC	3.79	1.53E-04	0.09
	LEKR1	-3.77	1.62E-04	0.1
Visceral Omentum Adipose	*CYP19A1*	6.40	1.51E-10	1.95E-06
	SNX11	4.86	1.19E-06	7.71E-03

Tissue	Gene	Z-score	P value	FDR
	AC004477.1	4.59	4.50E-06	0.02
	EVI2A	-4.58	4.73E-06	0.02
	NF1	4.34	1.41E-05	0.03
	NFATC2IP	4.31	1.66E-05	0.03
	AC004477.3	-4.30	1.71E-05	0.03
	EIF3C	4.29	1.83E-05	0.03
	AC109460.2	4.25	2.11E-05	0.03
	AC064836.2	4.08	4.49E-05	0.05
	ATXN2-AS	-4.08	4.58E-05	0.05
	BRD3	-3.98	6.82E-05	0.07
	CBX1	3.93	8.39E-05	0.08
Ovary	*HEY2*	-5.82	5.89E-09	5.07E-05
	EIF2AK4	5.09	3.64E-07	1.57E-03
	AC021755.3	4.92	8.61E-07	2.47E-03
	AC021755.2	4.74	2.09E-06	4.50E-03
	SNX11	4.64	3.43E-06	5.90E-03
	AC004477.1	4.56	5.20E-06	7.47E-03
	EIF3C	4.51	6.56E-06	8.07E-03
	AC004477.3	-4.40	1.06E-05	0.01
	EPHB6	-4.06	4.90E-05	0.05
	THBS1	3.93	8.41E-05	0.07
	ADSSL1	-3.86	1.14E-04	0.08
	FPGS	-3.85	1.16E-04	0.08
	UQCC1	-3.80	1.44E-04	0.1
Uterus	*AC021755.3*	4.78	1.77E-06	0.01
	SNX11	4.49	7.15E-06	0.02
	GDF5	4.40	1.09E-05	0.02

Tissue	Gene	Z-score	P value	FDR
	EIF3C	4.31	1.66E-05	0.02
	AC004477.3	-4.30	1.68E-05	0.02
	EIF2AK4	4.08	4.46E-05	0.05
	LEKR1	-4.07	4.69E-05	0.05
	CBX1	3.90	9.69E-05	0.08
	NAA60	3.88	1.06E-04	0.08
	ADSSL1	-3.85	1.18E-04	0.08
Vagina	*EIF2AK4*	5.31	1.07E-07	7.69E-04
	EEFSEC	4.61	4.00E-06	0.01
	SNX11	4.52	6.14E-06	0.01
	AC004477.3	-4.34	1.46E-05	0.03
	AC109460.2	4.22	2.42E-05	0.03
	ATF7IP2	-4.22	2.43E-05	0.03
	NFATC2IP	4.20	2.62E-05	0.03
	AL583810.1	-4.14	3.51E-05	0.03
	EVI2A	-4.14	3.52E-05	0.03
	AC021755.2	4.08	4.56E-05	0.03
	AC022296.4	-4.08	4.58E-05	0.03
	ADSSL1	-3.97	7.33E-05	0.04
	DKK1	3.91	9.20E-05	0.05
	UQCC1	-3.90	9.52E-05	0.05
	RPL6	-3.88	1.05E-04	0.05
	LEKR1	-3.86	1.14E-04	0.05
	EIF3C	3.84	1.21E-04	0.05
	RHOBTB2	-3.79	1.48E-04	0.06
	SLC23A3	-3.65	2.63E-04	0.1
	RNF103	3.65	2.66E-04	0.1

**Tissue**	**Gene**	**Z-score**	**P value**	**FDR**
Whole Blood	*CYP19A1*	6.03	1.61E-09	1.63E-05
	SNX11	4.73	2.22E-06	9.15E-03
	EEFSEC	4.66	3.20E-06	9.15E-03
	SKAP1	-4.63	3.61E-06	9.15E-03
	NPIPB6	4.56	5.00E-06	0.01
	AC004477.3	-4.39	1.12E-05	0.02
	TNFAIP8L3	4.20	2.72E-05	0.04
	SIVA1	-4.18	2.87E-05	0.04
	UQCC1	-4.16	3.21E-05	0.04
	NAA60	3.94	8.19E-05	0.08

Supplementary Table 7. Genes associated with endometrial cancer risk (FDR < 0.01), derived from S-MultiXcan analysis

Gene	Best performing tissue	N_{tissue}	P	FDR	Z_{min}	Z_{max}	Z_{mean}	Z_{SD}
GLDN	Adipose Subcutaneous	4	1.34E-12	1.77E-08	-2.02	3.65	-0.23	2.62
CYP19A1	Adipose Subcutaneous	2	9.52E-12	6.26E-08	5.75	6.17	5.96	0.3
SKAP1	Whole Blood	2	7.27E-09	3.19E-05	-6.11	-0.15	-3.13	4.22
HEY2	Ovary	1	9.94E-09	3.27E-05	-5.73	-5.73	-5.73	NA
SNX11	Adipose Subcutaneous	4	5.41E-07	1.42E-03	3.49	5.38	4.63	0.83
EEFSEC	Whole Blood	3	1.10E-06	2.42E-03	2.98	5.07	4.08	1.05
EVI2A	Whole Blood	3	1.50E-06	2.81E-03	-5.14	-3.45	-4.51	0.92
ATXN2L	Whole Blood	2	5.57E-06	9.15E-03	-3.87	-0.77	-2.32	2.19
SRP14	Adipose Visceral Omentum	3	6.62E-06	9.67E-03	-2.11	2.52	0.63	2.43
RHOBTB1	Adipose Visceral Omentum	2	9.11E-06	0.01	-1.55	4.51	1.48	4.28
RP5-890E16.5	Adipose Subcutaneous	4	9.19E-06	0.01	-4.72	-3.97	-4.37	0.32
ATF7IP2	Adipose Subcutaneous	1	1.39E-05	0.02	-4.35	-4.35	-4.35	NA
TSEN2	Adipose Subcutaneous	2	1.87E-05	0.02	-1.38	3.32	0.97	3.32
NFATC2IP	Vagina	3	2.05E-05	0.02	2.41	4.32	3.24	0.98
HMG20A	Adipose Subcutaneous	2	6.56E-05	0.06	2.62	3.84	3.23	0.86
HECTD4	Adipose Subcutaneous	1	9.29E-05	0.08	-3.91	-3.91	-3.91	NA
EIF2AK4	Adipose Visceral Omentum	6	0.00012	0.09	0.17	4.39	2.77	1.74
UQCC1	Adipose Subcutaneous	3	0.00012	0.09	-4.18	-3.18	-3.77	0.53
TMOD2	Whole Blood	3	0.00014	0.09	-2.08	0.12	-1.16	1.14

N_{tissue}: number of tissues available for this gene; FDR: false discovery rate; Z_{min}: minimum Z score from single tissue S-PrediXcan result; Z_{max}: maximum Z score from single tissue S-PrediXcan result; Z_{mean}: mean Z score from single tissue S-PrediXcan result; Z_{SD}: standard deviation of Z score from single tissue S-PrediXcan result.

Endometrial Cancer Association Consortium Collaborators

Frederic Amant[1], Daniela Annibali[1], Katie Ashton[2-4], John Attia[2, 5], Paul L. Auer[6, 7], Matthias W. Beckmann[8], Amanda Black[9], Louise Brinton[9], Daniel D. Buchanan[10-13], Stephen J. Chanock[14], Chu Chen[15], Maxine M. Chen[16], Timothy H.T. Cheng[17], Linda S. Cook[18, 19], Marta Crous-Bous[16, 20], Kamila Czene[21], Immaculata De Vivo[16, 20], Joe Dennis[22], Thilo Dörk[23], Sean C. Dowdy[24], Alison M. Dunning[25], Matthias Dürst[26], Douglas F. Easton[22, 25], Arif B. Ekici[27], Peter A. Fasching[8, 28], Brooke L. Fridley[29], Christine M. Friedenreich[19], Montserrat García-Closas[14], Mia M. Gaudet[30], Graham G. Giles[11, 31, 32], Dylan M. Glubb[33], Ellen L. Goode[34], Christopher A. Haiman[35], Per Hall[21, 36], Susan E. Hankinson[20, 37], Catherine S. Healey[25], Alexander Hein[8], Peter Hillemanns[23], Shirley Hodgson[38], Erling Hoivik[39, 40], Elizabeth G. Holliday[2, 5], David J. Hunter[16, 41], Angela Jones[17], Peter Kraft[16, 42], Camilla Krakstad[39, 40], Diether Lambrechts[43, 44], Loic Le Marchand[45], Xiaolin Liang[46], Annika Lindblom[47, 48], Jolanta Lissowska[49], Jirong Long[50], Lingeng Lu[51], Anthony M. Magliocco[52], Lynn Martin[53], Mark McEvoy[5], Roger L. Milne[11, 31, 32], Miriam Mints[54], Rami Nassir[55], Tracy A. O'Mara[33], Irene Orlow[46], Geoffrey Otton[56], Claire Palles[17], Paul D.P. Pharoah[22, 25], Loreall Pooler[35], Tony Proietto[56], Timothy R. Rebbeck[57, 58], Stefan P. Renner[59], Harvey A. Risch[51], Matthias Rübner[59], Ingo Runnebaum[26], Carlotta Sacerdote[60, 61], Gloria E. Sarto[62], Fredrick Schumacher[63], Rodney J. Scott[2, 4, 64], V. Wendy Setiawan[35], Mitul Shah[25], Xin Sheng[35], Xiao-Ou Shu[50], Melissa C. Southey[10, 31, 32], Amanda B. Spurdle[33], Emma Tham[47, 65], Deborah J. Thompson[22], Ian Tomlinson[17, 53], Jone Trovik[39, 40], Constance Turman[16], David Van Den Berg[35], Zhaoming Wang[9], Penelope M. Webb[66], Nicolas Wentzensen[9], Stacey J. Winham[67], Lucy Xia[35], Yong-Bing Xiang[68], Hannah P. Yang[9], Herbert Yu[45], Wei Zheng[50]

[1] Department of Obstetrics and Gynecology, Division of Gynecologic Oncology, University Hospitals KU Leuven, University of Leuven, Leuven, Belgium. [2] Hunter Medical Research Institute, John Hunter Hospital, Newcastle, New South Wales, Australia. [3] Centre for Information Based Medicine, University of Newcastle, Callaghan, New South Wales, Australia.

[4] Discipline of Medical Genetics, School of Biomedical Sciences and Pharmacy, Faculty of Health, University of Newcastle, Callaghan, New South Wales, Australia. [5] Centre for Clinical Epidemiology and Biostatistics, School of Medicine and Public Health, University of Newcastle, Callaghan, New South Wales, Australia. [6] Cancer Prevention Program, Fred Hutchinson Cancer Research Center, Seattle, WA, USA. [7] Zilber School of Public Health, University of Wisconsin-Milwaukee, Milwaukee, WI, USA. [8] Department of Gynecology and Obstetrics, Comprehensive Cancer Center ER-EMN, University Hospital Erlangen, Friedrich-Alexander-University Erlangen-Nuremberg, Erlangen, Germany. [9] Division of Cancer Epidemiology and Genetics, National Cancer Institute, Bethesda, MD, USA. [10] Department of Clinical Pathology, The University of Melbourne, Melbourne, Victoria, Australia. [11] Centre for Epidemiology and Biostatistics, Melbourne School of Population and Global Health, The University of Melbourne, Melbourne, Victoria, Australia. [12] Genomic Medicine and Family Cancer Clinic, Royal Melbourne Hospital, Parkville, Victoria, Australia. [13] University of Melbourne Centre for Cancer Research, Victorian Comprehensive Cancer Centre, Parkville, Victoria, Australia. [14] Division of Cancer Epidemiology and Genetics, National Cancer Institute, National Institutes of Health, Department of Health and Human Services, Bethesda, MD, USA. [15] Epidemiology Program, Fred Hutchinson Cancer

Research Center, Seattle, WA, USA. [16] Department of Epidemiology, Harvard T.H. Chan School of Public Health, Boston, MA, USA. [17] Wellcome Trust Centre for Human Genetics and Oxford NIHR Biomedical Research Centre, University of Oxford, Oxford, UK. [18] University of New Mexico Health Sciences Center, University of New Mexico, Albuquerque, NM, USA. [19] Department of Cancer Epidemiology and Prevention Research, Alberta Health Services, Calgary, AB, Canada. [20] Channing Division of Network Medicine, Department of Medicine, Brigham and Women's Hospital and Harvard Medical School, Boston, MA, USA. [21] Department of Medical Epidemiology and Biostatistics, Karolinska Institutet, Stockholm, Sweden. [22] Centre for Cancer Genetic Epidemiology, Department of Public Health and Primary Care, University of Cambridge, Cambridge, UK. [23] Gynaecology Research Unit, Hannover Medical School, Hannover, Germany. [24] Department of Obstetrics and Gynecology, Division of Gynecologic Oncology, Mayo Clinic, Rochester, MN, USA. [25] Centre for Cancer Genetic Epidemiology, Department of Oncology, University of Cambridge, Cambridge, UK.

[26] Department of Gynaecology, Jena University Hospital - Friedrich Schiller University, Jena, Germany. [27] Institute of Human Genetics, University Hospital Erlangen, Friedrich-Alexander University Erlangen-Nuremberg, Comprehensive Cancer Center Erlangen-EMN, Erlangen, Germany. [28] David Geffen School of Medicine, Department of Medicine Division of Hematology and Oncology, University of California at Los Angeles, Los Angeles, CA, USA. [29] Department of Biostatistics, Kansas University Medical Center, Kansas City, KS, USA. [30] Department of Population Science, American Cancer Society, Atlanta, GA, USA. [31] Cancer Epidemiology Division, Cancer Council Victoria, Melbourne, Victoria, Australia. [32] Precision Medicine, School of Clinical Sciences at Monash Health, Monash University, Clayton, Victoria, Australia. [33] Department of Genetics and Computational Biology, QIMR Berghofer Medical Research Institute, Brisbane, Queensland, Australia. [34] Department of Health Science Research, Division of Epidemiology, Mayo Clinic, Rochester, MN, USA. [35] Department of Preventive Medicine, Keck School of Medicine, University of Southern California, Los Angeles, CA, USA. [36] Department of Oncology, Södersjukhuset, Stockholm, Sweden. [37] Department of Biostatistics & Epidemiology, University of Massachusetts, Amherst, Amherst, MA, USA. [38] Department of Clinical Genetics, St George's, University of London, London, UK. [39] Centre for Cancer Biomarkers CCBIO, Department of Clinical Science, University of Bergen, Bergen, Norway.

[40] Department of Obstetrics and Gynecology, Haukeland University Hospital, Bergen, Norway. [41] Nuffield Department of Population Health, University of Oxford, Oxford, UK. [42] Program in Genetic Epidemiology and Statistical Genetics, Harvard T.H. Chan School of Public Health, Boston, MA, USA. [43] VIB Center for Cancer Biology, Leuven, Belgium. [44] Laboratory for Translational Genetics, Department of Human Genetics, University of Leuven, Leuven, Belgium. [45] Epidemiology Program, University of Hawaii Cancer Center, Honolulu, HI, USA.

[46] Department of Epidemiology and Biostatistics, Memorial Sloan-Kettering Cancer Center, New York, NY, USA. [47] Department of Molecular Medicine and Surgery, Karolinska Institutet, Stockholm, Sweden. [48] Department of Clinical Genetics, Karolinska University Hospital, Stockholm, Sweden. [49] Department of Cancer Epidemiology and Prevention, M. Sklodowska-Curie Cancer Center, Oncology Institute, Warsaw, Poland. [50] Division of

Epidemiology, Department of Medicine, Vanderbilt Epidemiology Center, Vanderbilt-Ingram Cancer Center, Vanderbilt University School of Medicine, Nashville, TN, USA. [51] Chronic Disease Epidemiology, Yale School of Public Health, New Haven, CT, USA. [52] Department of Anatomic Pathology, Moffitt Cancer Center & Research Institute, Tampa, FL, USA. [53] Institute of Cancer and Genomic Sciences, University of Birmingham, Birmingham, UK. [54] Department of Women's and Children's Health, Karolinska Institutet, Stockholm, Sweden. [55] Department of Biochemistry and Molecular Medicine, University of California Davis, Davis, CA, USA. [56] School of Medicine and Public Health, University of Newcastle, Callaghan, New South Wales, Australia. [57] Harvard T.H. Chan School of Public Health, Boston, MA, USA. [58] Dana-Farber Cancer Institute, Boston, MA, USA. [59] Department of Gynaecology and Obstetrics, University Hospital Erlangen, Friedrich-Alexander University Erlangen-Nuremberg, Comprehensive Cancer Center Erlangen-EMN, Erlangen, Germany. [60] Center for Cancer Prevention (CPO-Peimonte), Turin, Italy. [61] Human Genetics Foundation (HuGeF), Turino, Italy. [62] Department of Obstetrics and Gynecology, School of Medicine and Public Health, University of Wisconsin, Madison, WI, USA. [63] Department of Population and Quantitative Health Sciences, Case Western Reserve University, Cleveland, OH, USA. [64] Division of Molecular Medicine, Pathology North, John Hunter Hospital, Newcastle, New South Wales, Australia. [65] Clinical Genetics, Karolinska Institutet, Stockholm, Sweden. [66] Population Health Department, QIMR Berghofer Medical Research Institute, Brisbane, Queensland, Australia. [67] Department of Health Sciences Research, Division of Biomedical Statistics and Informatics, Mayo Clinic, Rochester, MN, USA. [68] State Key Laboratory of Oncogene and Related Genes & Department of Epidemiology, Shanghai Cancer Institute, Renji Hospital, Shanghai Jiaotong University School of Medicine, Shanghai, China.
