# Supplementary material for: Multi-tissue transcriptome-wide association study identifies eight candidate genes and tissue-specific gene expression underlying endometrial cancer susceptibility
Source: Commun Biol. 2021 Oct 21;4:1211. doi: 10.1038/s42003-021-02745-3 (PMC8531339; doi:10.1038/s42003-021-02745-3)
Supplement: Supplementary file 3 — Description of Additional Supplementary Files [file 42003_2021_2745_MOESM3_ESM.pdf]

Description of Additional Supplementary Files

File name: Supplementary Data 1

Description: Candidate endometrial cancer susceptibility gene expression data for cross-tissue correlations (Figure 1).
